# Supplementary figures and images for: Characterization of the Bacterial Composition of 47 Fermented Foods in Sweden
Source: Foods. 2023 Oct 19;12(20):3827. doi: 10.3390/foods12203827 (PMC10606000; doi:10.3390/foods12203827)

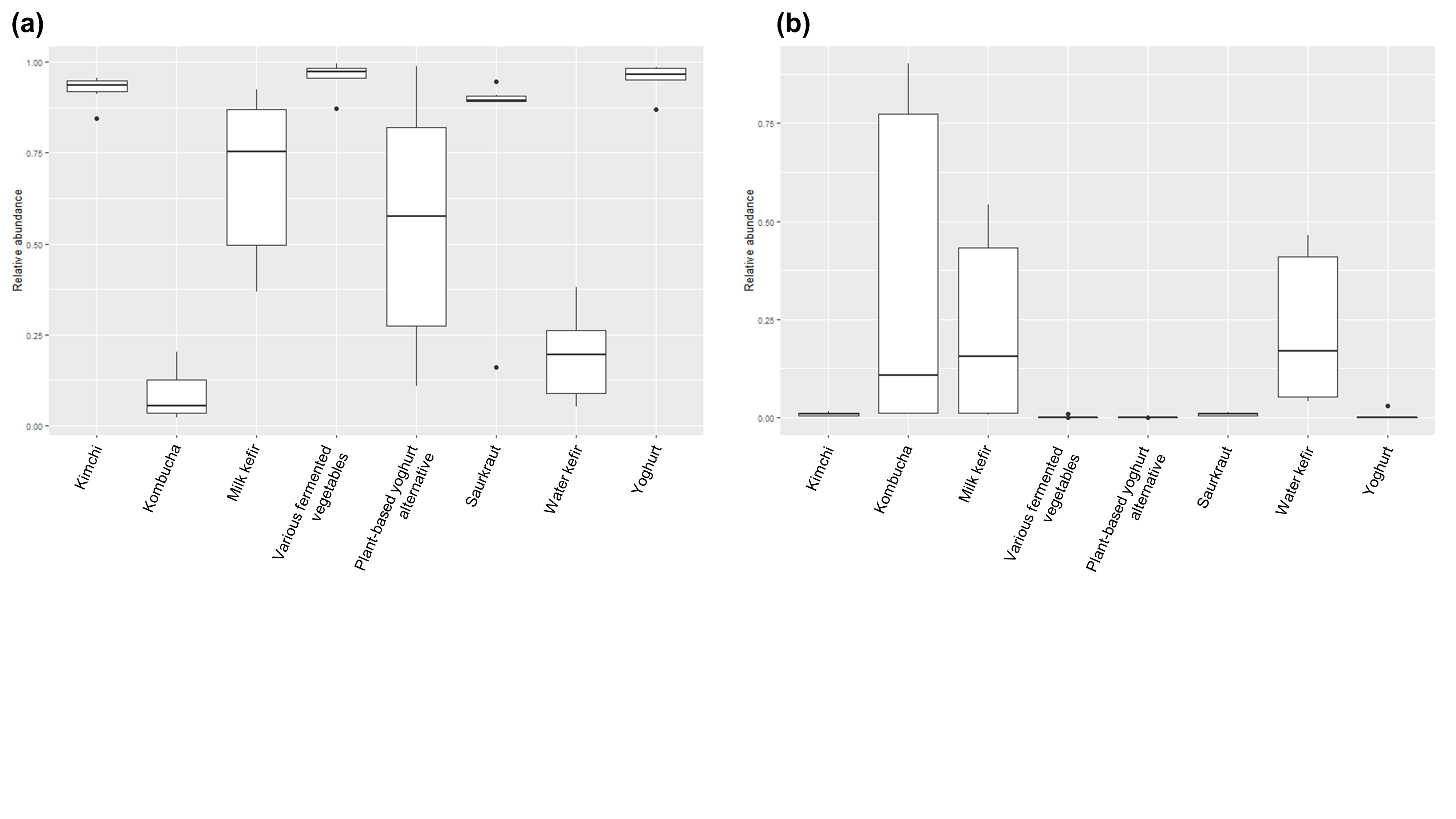

Supplement: Supplementary file 1 [file foods-12-03827-s001.zip › Figure S1.PNG]
